# Sepsis care protocol: initial evaluation at a university hospital in southern Brazil

**DOI:** 10.1186/cc12944

**Published:** 2013-11-05

**Authors:** Fabiano Ramos, Marilaine P Silva, Paola H Alves, Michèle S Borges, Miriane MS Moretti, Georgia L Silva, Leticia G Lobo, Silvia PT Soares

**Affiliations:** 1 Hospital São Lucas, Pontifícia Universidade Católica Rio Grande do SUL, Porto Alegre, Brazil

## Background

Sepsis is a systemic inflammation caused by severe infection. It is a life-threatening condition, progresses rapidly, and affects multiple system functions. An evidence-based medical sepsis bundles model has been used for sepsis care in clinical practice. All patients who have at least two signs or symptoms of systemic inflammatory response syndrome (SIRS) secondary to an infectious process are considered septic. Sepsis is the leading cause of death in ICUs and a major cause of late hospital mortality rate, exceeding the acute coronary syndromes and neoplasms. Mortality in Brazil reaches 60%, while the world average is around 30%, overcoming countries such as India and Argentina. The early recognition and treatment of these patients are key to reducing mortality. The aim of this study is to evaluate the implementation of the protocol of sepsis in a university general hospital in Porto Alegre.

## Materials and methods

Retrospective evaluation of protocols for sepsis in emergency in 2012.

## Results

A total of 200 patients were enrolled in the protocol during the study period. The average age was 35 years (SD ± 16.5), 51% of patients were male, the most frequent focus was respiratory 61%, and the second urinary with 14%. Clinical criteria for inclusion in the protocol that most prevailed were: axillary temperature and heart rate, with more than 95%. Altered axillary temperature was present in 98% of the sample. Of these cases, 86.5% (*n *= 173) of patients were discharged within 24 hours. Twenty-seven patients met criteria for hospitalization, 22% required the ICU. Around 75% (*n *= 20) of inpatients had no blood cultures collected before starting antibiotics. Only 7% mortality (*n *= 2).

## Conclusions

The criteria for inclusion in the protocol are quite sensitive and the number of visits per month in the emergency exceeds 10,000. A total of 200 patients enrolled to the sepsis care protocol in a year, over 80% of these being discharged within 24 hours, suggests a low adherence to institutional protocol, especially in patients with septic shock, which is reinforced by the very low mortality compared with literature data. The evaluation of these data was essential to bring the knowledge that adherence to the protocol is still very low in our institution.

